# Descriptors of multidrug-resistant TB deaths in Ethiopia

**DOI:** 10.5588/pha.23.0030

**Published:** 2023-12-07

**Authors:** E. Tesema, Z. G. Dememew, D. G. Datiko, A. Gebreyohannes, Y. Molla, A. Tefera, G. Gizatie, T. Bogale, M. Million, P. G. Suarez, M. M. Aseressa, D. Jerene, M. Biru

**Affiliations:** 1 United States Agency for International Development (USAID) Eliminate TB Project, KNCV, Addis Ababa, Ethiopia; 2 USAID Eliminate TB Project, MSH, Addis Ababa, Ethiopia; 3 Management Sciences for Health, Global Health Systems Innovation, Arlington, VA, USA; 4 KNCV Tuberculosis Foundation, The Hague, The Netherlands

**Keywords:** Ethiopia, drug-resistant TB deaths, rifampicin-resistant TB, RR/MDR-TB, TB-related mortality

## Abstract

Deaths related to multidrug-resistant TB among patients who had received a second-line anti-TB drugs in Ethiopia were analysed. Respectively 38/704 (5.4%) and 44/995 (4.4%) deaths were identified in two cohorts (2015 and 2022). In the 2015 cohort, severe malnutrition was less prevalent, previous treatment rates were three times higher, hypokalaemia was more frequent, and the use of the Xpert® MTB/RIF assay, respiratory failure and severe anaemia/pancytopenia were less common than in the 2022 cohort. We observed that there were variations in adverse events when different treatment regimens were used over different time periods. To ensure proper patient care, correct guidance must be consistently implemented.

## Abstract (fr)

Les décès liés à la TB multirésistante chez les patients ayant reçu des médicaments antituberculeux de seconde ligne en Éthiopie ont été analysés. Respectivement 38/704 (5,4%) et 44/995 (4,4%) décès ont été identifiés dans deux cohortes (2015 et 2022). Dans la cohorte 2015, la malnutrition sévère était moins fréquente, les taux de traitement antérieur étaient trois fois plus élevés, l’hypokaliémie était plus fréquente, et l’utilisation du test Xpert^®^ MTB/RIF, l’insuffisance respiratoire et l’anémie/pancytopénie sévère étaient moins fréquentes que dans la cohorte 2022. Nous avons observé des variations dans les effets indésirables lorsque différents schémas thérapeutiques étaient utilisés sur différentes périodes. Pour garantir des soins adéquats aux patients, des consignes appropriées doivent être appliquées de manière régulière.

Drug-resistant TB (DR-TB) remains a critical concern for public health. According to 2019 data from Ethiopia, an estimated 1.1% of the new TB cases and 7.5% of those who had previously undergone TB treatment among the reported TB cases were found to have rifampicin-resistant TB (RR-TB).^1^,^2^ In 2020, Ethiopia was removed from the list of 30 countries with a high burden of rifampicin-resistant/multidrug-resistant TB (RR/MDR-TB).^2^

COVID-19 pandemic has reversed years of progress in providing essential TB services, including the reduced numbers of TB notifications, access to TB diagnostics tests, enrolment for effective treatments and provision of quality services.^1^ The overall number of TB deaths have also increased due to reduced access to TB diagnosis and treatment because of the pandemic.^1^ This may have also had adverse consequences for DR-TB mortality. Improving patient-centred care delivery to DR-TB patients by implementing regular clinical analysis of unfavourable outcomes, specifically, DR-TB-related deaths helps to identify common cause of deaths that could guide the development of targeted interventions and provide practical recommendations to healthcare workers (HCWs) in the field.

## METHODS

### Study area, period, population, and data collection procedure

A hospital-based review was conducted in selected high-load RR/MDR-TB treatment initiating centres (TICs). We analysed RR/MDR-TB patient charts and registers using structured checklists to describe the case fatality of RR/MDR-TB, the distribution and magnitude of clinical parameters related to RR/MDR-TB deaths. Data were collected from June 2012 to June 2015 (the 2015 cohort) from Amhara and Oromia Regions; and from July 2020 to June 2022 (the 2022 cohort) from the Oromia, Amhara, Addis Ababa, South Nation and Nationalities and Peoples (SNNP) Regions to compare relevant variables related to TB deaths. Sociodemographic variables (age, sex), HIV status, previous TB treatment and comorbid diseases were some of the variables included in the analysis. All deaths in selected treatment centres were line-listed and their charts were reviewed to get the necessary information.

We conducted descriptive statistical analyses. This review was carried out after obtaining approval for the extraction of the secondary data from the regional ethical clearance committee.

## ASPECTS OF INTEREST

### Sociodemographic and clinical characteristics

In the 2015 cohort of patients, a total of 38 MDR/RR-TB deaths out of a total of 704 RR/MDR-TB patients were documented in Oromia and Amhara Regions of the country. In the 2022 cohort of patients, a total of 44 MDR/RR-TB deaths out of a total of 995 RR/MDR-TB patients were registered and reviewed from Addis Ababa, SNNP, Oromia and Amhara. RR/MDR-TB-related death rates were 5.4% in the 2015 cohort and 4.4% in 2022 cohort. In the 2022 cohort, more than 93% of patients had severe form of disease at the time of diagnosis, which may have contributed to early death. Compared to the 2022 cohort, severe malnutrition was less prevalent (odds ratio [OR] 0.24, 95% confidence interval [CI] 0.93–0.63), history of previous treatment was three times higher (OR 2.77, 95% CI 1.05–7.24), electrolyte imbalance (hypokalaemia) was more frequent (28% vs. 3%, Fisher’s Exact test,*P* = 0.001), and Xpert^®^ MTB/RIF (Cepheid, Sunnyvale, CA, USA) testing (37% vs. 95%, *P* < 0.001), respiratory failure (3% vs. 29%, Fisher’s Exact test, *P* = 0.013) and severe anaemia/pancytopenia were less common (3% vs. 35%, Fisher’s Exact test, *P* = 0.003) in the 2015 cohort. Early death (within 2 months of treatment initiation) and other factors such as comorbidities (HIV and diabetes mellitus) in the two cohorts were similar (Table). In the 2022 cohort, 41/44 (93%) of patients who died had severe forms of TB (mainly disseminated TB, miliary TB and extensive bilateral lung ­disease) at the time of diagnosis (Figure).

**TABLE:** Sociodemographic and clinical data among RR/MDR-TB patients who died during the review period in 2015 and 2022

Variables	2015 (*n* = 38) *n* (%)	2022 (*n* = 44) *n* (%)	OR (95% CI)	*P* value
Male sex	16 (43)	25 (57)	0.58 (0.24–1.39)	0.224
Mean age, years	37.1	38.7		
Severe malnutrition	8 (39)	23 (51)	0.24 (0.93–0.63)	0.004*
Early death, less than 2 months	31 (80)	40 (93)	—	0.331
HIV-positive	10 (30.3)	12 (27.9)	0.98 (0.36–2.60)	0.980
DM	3 (14)	4 (15)	—	1.000
Confirmed using Xpert MTB/Rif	14 (37)	42 (95)	—	<0.001*
Confirmed using culture/LPA	21 (60)	2 (5)	—	—
Missed	3	0		
Previous treatment history	32 (84)	26 (61)	2.77 (1.05–7.24)	0.038*
Home death	23 (42.4)	20 (45.5)	1.84 (0.77–4.41)	0.173
Adverse events	30 (66)	31 (70.4)	1.57 (0.58–4.24)	0.379
Hypokalaemia	11 (28)	1 (3)	—	0.001*
Renal failure/uraemia	4 (13)	2 (6)	—	0.424
Refractory vomiting/severe dyspepsia	5 (17)	2 (6)	—	0.255
Hepatitis	3 (10)	1 (3)	—	0.354
Severe anaemia/pancytopenia	1 (3)	11 (35)	—	0.003*
Psychosis	2 (7)	4 (13)	—	0.671
Respiratory failure	1 (3)	9 (29)	—	0.013*
Congestive heart failure	1 (3)	1 (3)	—	1.000

*Statistically significant.

RR/MDR-TB = rifampicin-resistant/multidrug-resistant TB; OR = odds ratio; CI = confidence interval; DM = diabetes mellitus; LPA = line-probe assay.

**FIGURE:**
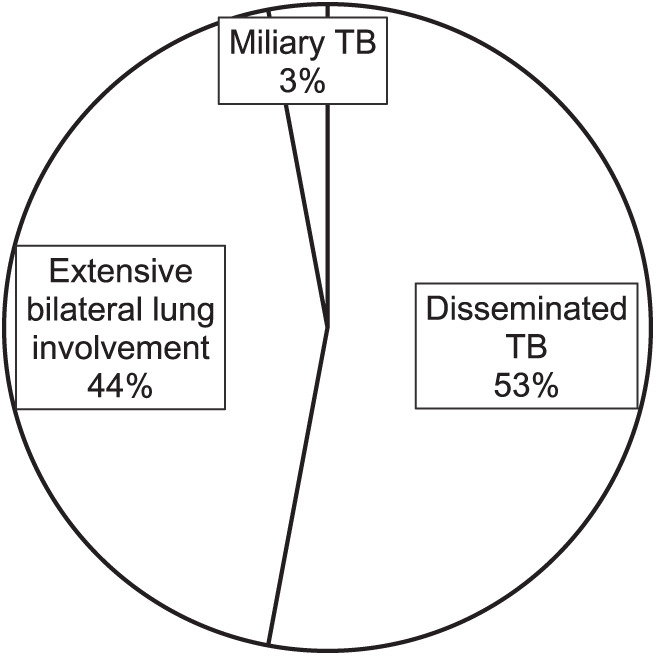
RR/MDR-TB disease severity status in 2022 cohort (*n* = 44). RR/MDR-TB = rifampicin-resistant/multidrug-resistant TB.

In the 2015 cohort, the standard long treatment regimen that included injectables was the predominant treatment regimen, with over 95% of patients receiving it in all TICs across the country. In the 2022 cohort, the all-oral bedaquiline (BDQ) and linezolid (LZD) containing longer treatment regimens were administered to 57% of patients, followed by the short, all-oral, BDQ-containing regimen (23%). The remainder (16%) were on individualised regimens. Only 5% of patients were on injectable-containing treatment regimens. RR/MDR-TB patients were managed in line with Ethiopian national guideline recommendations for the management of RR/MDR-TB.^2^

## DISCUSSION

Our review indicates that RR/MDR-TB-related mortality is more prevalent in patients with malnutrition, which has been worsening over time. Malnutrition was observed in individuals with severe disease and typically manifested within the initial months of presentation during the study periods. Almost all patients had severe forms of the disease at the time of diagnosis, which may have contributed to early death. Although the use of molecular diagnostic testing (GeneXpert) has increased, the rate of early deaths remained similar in the two cohorts. Risk factors such as previous treatment and comorbidities like malnutrition, diabetes mellitus and HIV/AIDS, were frequent in both cohorts, which is in concordance with other studies.^3^–^6^ We identified variations in adverse events, including the shift from an injectable-based regimen with notable electrolyte imbalances in the 2015 cohort to an all-oral BDQ and LZD-based regimen with a significant occurrence of severe anaemia/pancytopenia in the more recent 2022 cohort. These variations in adverse events are consistent with reports from other countries.^7^,^8^

## CONCLUSION

This hospital-based TB mortality review showed severe malnutrition was more prevalent among patients who died of RR/MDR-TB. The persistence of early deaths underscores the need for additional prospective studies to gain a clearer understanding of the underlying causes of these fatalities. Patients experienced adverse events while on treatment, and these varied according to regimen type and the presence of risk factors like severe malnutrition and a history of previous treatment. We strongly recommend adjunctive nutritional care, including therapeutic feeding, for all DR-TB patients as standard of care. We also recommend the use of emergency care management services for RR/MDR-TB patients to prevent early death. Similarly, patients experiencing adverse events including drug toxicities, those with comorbid conditions and risk factors should be managed in consultation with senior experts based on guidelines and global recommendations. We also recommend implementing community awareness programmes that promote psychosocial support and encourage early healthcare-seeking behaviour.
